# Effects of wingbeat kinematics and muscle size on maximum flight speed in hummingbirds

**DOI:** 10.1242/jeb.251012

**Published:** 2026-08-21

**Authors:** Nadje A. Najar, Sean C. Wilcox, Leanna R. Fernandez, Christopher J. Clark

**Affiliations:** ^1^ Department of Evolution, Ecology, and Organismal Biology, Spieth Hall, University of California, Riverside, Riverside, CA 92521, USA; ^2^ Biological Sciences Department, Moorpark College, Moorpark, CA 93021, USA; ^3^ School of Biological Sciences, Zoology Building, University of Aberdeen, Aberdeen, AB24 2TZ, Scotland, UK

**Keywords:** Sexual dimorphism, Burst capacity, Wingbeat kinematics, Wind tunnel, Mixed model, Path analysis, Asymptotic load lifting, Flight performance

## Abstract

Flight speed has important fitness consequences for flying animals, but how the parts of the flight apparatus limit top speed remains unknown. Morphology, kinematics and flight performance are strongly correlated with species and sex class, which complicates attempts to dissect the contribution of each element to flight performance. We performed two experiments to assess how sexual dimorphism within a species and morphological variation between species influence maximum flight speed. Burst capacity is often hypothesized to be representative of generalized flight performance and may be correlated with top speed. In experiment one, we found top speed and burst capacity were negatively correlated in female but not male *Archilochus alexandri*. In experiment two, we used path analysis to disentangle the relative contributions of muscle size and wingbeat kinematics on maximum flight speed in both sexes of four species: *A. alexandri*, *Calypte anna*, *Calypte costae* and *Selasphorus sasin*. Considered separately, muscle size was more strongly associated with variation in both flight speed and burst capacity compared with wingtip velocity. In a *post hoc* model comparing muscle size and wingbeat kinematics, only muscle size remained a significant predictor for both assays, but substantial variance in top speed remains unexplained. There was no significant correlation between flight speed and burst capacity despite sex-specific differences, suggesting flight muscle composition may be an important predictor of performance for a given flight assay. Overall, top speed and burst capacity represent uncorrelated measures of flight ‘performance’, and significant predictors of flight speed remain unexplored.

## Teaser

**Summary:** Muscle size is better than wing kinematics in explaining variation in maximal forward flight speed and load lifting capacity in both male and female hummingbirds.

## INTRODUCTION

**Fig. 1.**
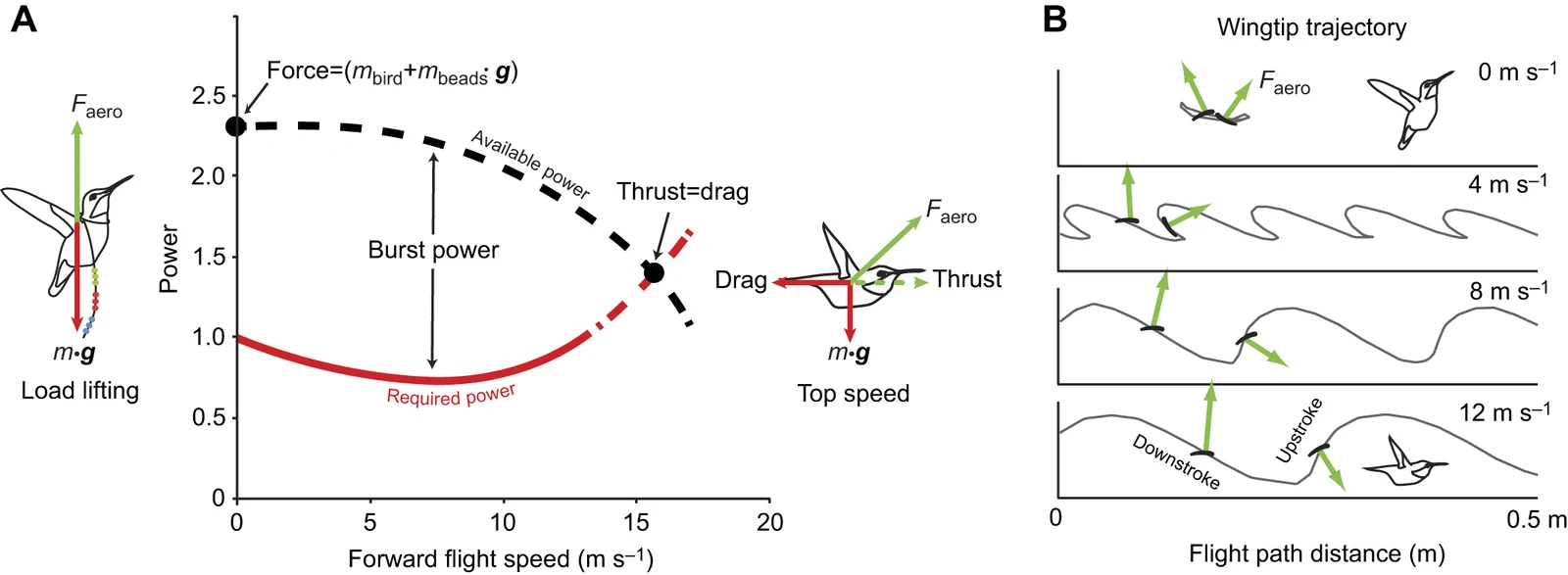
**Limits to flight performance in hummingbirds.** (A) The ‘burst power’ available for flight maneuvers is a function of forward flight speed, illustrated with empirical data for male Anna's hummingbirds (*Calypte anna*). The solid red line indicates the power required for steady flight. The empirical metabolic power curve (*V̇*_O_2__) is shown normalized to the power required to hover (from figure 1A in Clark and Dudley, 2010; for airspeeds from 0 to 13 m s^−1^). Extrapolating beyond the end of the regression to the empirically measured top speed in a wind tunnel, 15.4 m s^−1^ (from Clark and Dudley, 2009) (dashed red line), predicts that the available power at 15.4 m s^−1^ is 1.5 times the power required to hover. The black dashed line depicts a hypothesis of the available burst power. Neither assay used in this paper measures power; rather, they measure force balance. The load lifting assay tests the maximum force (*F*_aero_) an animal can transiently produce, for a few seconds, at low speed (0 m s^−1^). For male *C. anna*, *F*_aero_ is approximately 2.3 times their body weight (data from Segre et al., 2015). If muscle power scales proportionately to force (Chai and Millard, 1997), the available power is 2.3 times the power required to hover. Top speed is reached when the animal's physical capacity to produce aerodynamic thrust is equal and opposite to total drag. The top speed assay used in this study takes 1–2 min to perform, i.e. under conditions that are less transient than the load lifting assay. ‘Burst power’, the difference between the red curve and the black curve, is the excess power available for accelerating (maneuvers). The black dashed line is for illustrative purposes only and must not be considered as an actual prediction, as the functional form of available power between 0 m s^−1^ and top speed is currently unknown. (B) Lateral view of wingtip paths during hovering (0 m s^−1^) and forward flight of a female rufous hummingbird (*Selasphorus rufus*) (4, 8, 12 m s^−1^) (from figure 7 in Tobalske et al., 2007). As flight speed increases, the advance ratio increases; thus, the path of the wingtip trajectory flattens. As a result, the capacity to produce thrust diminishes, because the aerodynamic force vector has less capacity to point forward, e.g. during upstroke. Green vectors are for illustration only and are not derived from actual data.

Maximum flight speed plays a major role in an animal's ecology. Top speed is critical for certain behaviors with high fitness consequences: predator evasion, prey capture, competition with conspecifics and courtship (Tobalske et al., 2007; Clark, 2009; Combes et al., 2012). Hummingbirds are capable of reaching translational velocities of 13–15 m s^−1^ in level horizontal flight (Gill, 1985; Chai et al., 1999; Clark and Dudley, 2009), and exceed 20 m s^−1^ in gravity-assisted dives performed as a part of courtship displays (Pearson et al., 1960; Clark, 2009). Hummingbirds are also adept at hovering in place (flying at 0 m s^−1^) while visiting a flower or feeder (Warrick et al., 2005). The hummingbird flight phenotype is therefore adapted for both low-speed and high-speed flight. While both slow and fast flight are ecologically relevant, and slow flight (<6 m s^−1^) has been the subject of dozens of studies, the top speed of hummingbirds in a calibrated variable airspeed wind tunnel has been described in only three studies (Chai and Dudley, 1999; Chai et al., 1999; Clark and Dudley, 2009). During level (horizontal) flight, flapping must produce enough forward force (thrust) to overcome drag (Fig. 1A). The bird will accelerate until thrust is equal and opposite to total drag force, at which point it has reached top speed. However, how top speed is limited by morphological and kinematic variation in birds is poorly understood.

During flapping flight, faster forward flight speed is associated with increases in wingtip velocity resulting from higher stroke amplitudes and wingbeat frequencies (Tobalske et al., 2003, 2007). Hummingbirds sustain their characteristically high wingbeat frequencies (ranging from 12 to 100 Hz) (Greenwalt, 1960; Wilcox and Clark, 2022) via early activation of each flight muscle before wingtip reversal (Ingersoll and Lentink, 2018). This mode of overlapping neuromuscular activation may constrain wingbeat frequency to a preferred resonance window to avoid tetanus (Tobalske et al., 2010), and wingbeat frequency is observed to change little across a wide range of flight muscle power outputs (Altshuler, 2006; Tobalske et al., 2007). Modulation of stroke amplitude is therefore the more likely way hummingbirds increase wingtip velocity. Hummingbirds have been observed to briefly exceed a stroke amplitude of 180 deg while under maximal loading while hovering (Chai and Dudley, 1995; Chai et al., 1996; Altshuler and Dudley, 2003). The additional muscle power required to maintain a constant wingbeat frequency is likely supplied by the recruitment of motor units, as the amplitude of electromyogram spike measurements is correlated with stroke amplitude and flight speed (Tobalske et al., 2010). Drag is proportional to velocity squared (Norberg, 1995), so the power required to fly at faster forward speeds rises rapidly. For a given wing, maximum forward flight speed may therefore be limited primarily by available muscle power, with the fastest individuals being those with large enough flight muscles to maximize stroke amplitude, hereafter termed the muscle size hypothesis.

Flight kinematics may limit thrust production before maximum muscle power output can be reached. Fixed-pitch aircraft propellers, for instance, produce high thrust only for a specific window of flight speeds, as characterized by the advance ratio (Sechler and Dunn, 1942). The advance ratio is airspeed relative to wingtip velocity (Song et al., 2016), which in birds varies between an advance ratio of 0 (hovering) to about 3 in high speed flight (figure 14 in Song et al., 2016). As the advance ratio increases, the trajectory of the wingtip, in a global frame of reference, gradually flattens, especially during the downstroke (Fig. 1B; Tobalske et al., 2007). Song et al. (2016) showed that thrust production becomes prominent in the upstroke in a calliope hummingbird (*Selasphorus calliope*) flying at an intermediate speed (8.3 m s^−1^). Top speed may therefore be limited by wing kinematics, such as the proportion of time the wing spends in translation during the upstroke or the rotation speed of the wings during stroke reversal. Wing inertia is lowest in shorter, more pointed wings, and wingbeat frequency is strongly correlated with wing length in hummingbirds. Birds with the shortest wings and highest wingbeat frequencies may be able to achieve greater increases in wingtip velocity and advance ratio before the maximum stroke plane angle is achieved, resulting in higher top speeds, hereafter termed the wing kinematics hypothesis.

One of the key differences between the muscle size and kinematics hypotheses is the expected relationship between top speed and maximal muscle power output. In hummingbirds, muscle power is commonly measured with a load lifting assay where a string of beads of known mass is attached to a bird induced to fly straight up. The total mass the hummingbird can lift at the apogee of its vertical flight is equal and opposite to the amount of aerodynamic force generated by the wings and muscles. Under conditions of maximal loading, hummingbirds increase aerodynamic force by increasing stroke amplitude, approaching the anatomical limit of ∼180 deg in which the wingtips of the opposing wings collide, while maintaining or slightly increasing their wingbeat frequency (Chai and Millard, 1997). The burst power measurement derived from the load lifting assay has previously been interpreted as a proxy for overall hummingbird flight performance ability (Segre et al., 2015; Dakin et al., 2020). If available power from muscles is the main limitation on top speed, then we would expect top speed to be positively related to load lifting ability. Conversely, if wingbeat kinematics is the main limitation, we would expect to observe a non-significant or even negative relationship between load lifting ability and top speed. For example, flight performance metrics may instead represent a trade-off: longer wings with lower wing loadings improve load lifting capacity, but shorter, higher loading wings are favored for fast flight (Norberg, 1995). This scenario also implies muscle power output at top speed is not as high as during load lifting.

We conducted two experiments to test our hypotheses on the morphological and kinematic correlates of top speed. First, we compared a sample of 53 male and female black-chinned hummingbirds (*Archilochus alexandri*) to investigate how sexual dimorphism in phenotype and load lifting ability is correlated with differences in flight speed. This experiment revealed sex differences in flight performance. However, as the morphological differences between male and female *A. alexandri* are statistically confounded by sex, this initial experiment did not entirely address our wing kinematics and muscle size hypotheses, as other un-measured sexual differences could potentially drive the differences in performance that we observed. Therefore, in a second experiment, we measured top speed in an additional 44 adult male and female hummingbirds in four species: *A. alexandri*, Anna's hummingbird (*Calypte anna*), Costa's hummingbird (*Calypte costae*) and Allen's hummingbird (*Selasphorus sasin*). Each species differs in morphology and flight kinematics, and all four are sexually dimorphic in body mass, wing length and keel length (Table 1). This variation by species and sex class allowed us to disentangle the effect of each component of the flight apparatus on top speed to test our muscle size and wing kinematics hypotheses. We used path analysis, a simplified form of structural equation modeling, to simultaneously estimate the direct and indirect effects of multiple independent predictor variables on our dependent variables, top speed and load lifting ability (Wright, 1934; Streiner, 2005; Collins and Higham, 2017). We tested our wing kinematics and muscle size hypotheses *a priori* by comparing the performance of two path models in explaining variation in top speed. We also used these models to test the hypothesis that either top speed or load lifting can be used as a proxy of generalized flight performance.

**Table 1.** Morphological variation in mass, folded wing length and keel length among males and females of four hummingbird species

Species	Sex	*N*	Mass (g)	Rank	Wing length (mm)	Rank	Keel length (mm)	Rank
*C. anna*	♂	6	4.52±0.36	1	48.19±1.47	2	19.86±0.23	1
	♀	10	4.01±0.29	2	48.36±1.73	1	17.94±0.39	2
*S. sasin*	♂	5	3.46±0.34	4	37.74±0.97	8	17.77±0.3	3
	♀	6	3.55±0.15	3	41.92±1.14	6	17.55±0.65	4
*C. costae*	♂	7	3.15±0.14	6	43.27±1.5	5	16.97±0.28	5
	♀	6	3.03±0.17	8	44.62±1.72	4	16.3±0.51	6
*A. alexandri*	♂	31	3.09±0.32	7	40.5±0.85	7	16.26±0.58	7
	♀	22	3.42±0.21	5	45.0±1.4	3	15.6±0.52	8

Data are shown for Allen's hummingbird (*Selasphorus sasin*), Anna's hummingbird (*Calypte anna*), Costa's hummingbird (*Calypte costae*) and black-chinned hummingbird (*Archilochus alexandri*). *N* is the number of birds. Mass, wing and keel data are means±s.d. Ranking of each species–sex class (where 1 is largest/longest and 8 is smallest/shortest) depends on the character. Thus, which species–sex class is predicted to be fastest or to lift the largest loads will depend on which character limits each assay the most.

## MATERIALS AND METHODS

### General methods

Data were acquired from two separate experiments with significant overlap in methods. Their common features are described here. Wild hummingbirds were captured using feeder traps in Riverside, CA, USA, and scored for folded wing length, keel length and wing feather molt. Hummingbirds were transported to UC Riverside after capture. Five minutes before flight performance trials, each bird was fed a meal of 30% (w/w) sugar water for 3 s to diminish any effect of hypoglycemia, as birds captured at feeders are presumably hungry. Birds were subjected to two flight assays (top speed and load lifting, described below), banded with an aluminium USGS band, marked with white-out to avoid recapture, then released the same day they were captured.

The protocol to measure top speed was similar to that in Clark and Dudley (2009). Top speed was measured by placing a bird in the working section (94 cm×36 cm×36 cm) of a variable speed wind tunnel (described in Clark and Mistick, 2018). Wind speed was calibrated using a pitot tube and hot wire anemometer. Before each trial, birds were offered a 3 s sucrose meal, weighed to the nearest 0.01 g, and placed in the tunnel. The starting airspeed was set to 9 m s^−1^, then accelerated by approximately 0.05 m s^−2^ while the investigator stood near the back of the working section (downwind) to encourage the bird to fly at the front of the working section. When the bird was no longer able to maintain its forward position and drifted downwind back past a predetermined set point (∼30 cm from the back), the air was switched off and the airspeed recorded. On rare occasions, birds hit the back mesh (15 mm square deer netting) of the working section, although the deceleration of the windspeed normally precluded this. When this occurred, the bird was allowed to perch with the wind off and preen its feathers for 1 min before commencing the next trial. This assay was performed multiple times consecutively with a short rest period in between each trial.

Asymptotic load lifting was measured followed published methods (Chai and Millard, 1997; Chai et al., 1997). This assay measures birds' ability to produce upwardly directed aerodynamic force (*F*_aero_ in Fig. 1) at flight speeds near 0 m s^−1^ and is therefore an estimate of flight muscle burst capacity. Briefly, we placed a small rubber band attached to string of colored beads around the bird's neck. We then placed the bird on the floor of a flight chamber (35×35×62 cm) with darkened sides and an open, light top, which induces the bird to fly upward to escape. As the bird flies upward, it lifts incrementally more beads until it reaches a zenith and can no longer ascend. We used video recordings (Canon Vixia HF R500; 30 Hz) to determine the maximum number of beads lifted at the zenith of each trial (Movie S1). Total mass lifted was calculated by adding the mass of the beads lifted to the pre-trial mass of the bird. As in the top speed assay, each bird performed several trials consecutively with a short rest period between.

### Experiment 1: sexual dimorphism in *A. alexandri*

*Archilochus alexandri* (Bourcier & Mulsant 1846) were captured between June and late August of 2015 and 2016, which corresponds to midway through the breeding season (June) to after the breeding season but before they began fattening for migration (August). In 2015, birds experienced the two flight assays described above (high-speed flight in a wind tunnel and asymptotic load lifting), as well as a third assay – flight through a string maze – performed in a random order. In 2016, we only subjected them to the two flight assays described above. The string maze assay was an attempt to quantify maneuverability. However, repeatability (intraclass correlation) of the two maze performance variables we measured was lower (0.61 for time taken to traverse the maze and 0.17 for number of strings hit within the maze) than that for the other two assays (>0.8). Therefore, we do not present any data for it, and we abandoned the string maze assay in 2016. We mention it here because the string maze assay took longer to conduct (∼15 min) than the other flight assays, and the random order of presentation meant we had to statistically account for the possibility of fatigue effects in the birds that performed it first.

The flight performance assays were presented in random order, with 5 min of rest between each assay to minimize any carry-over effect of fatigue between assay performances. Each bird (*N*=53) performed four consecutive trials per assay. We discarded each bird's slowest speed and lowest load lifting trials, leaving three trials per assay for use in statistical analyses. We allowed 1 min of rest between trials of high-speed flight in the wind tunnel and load lifting. We analyzed order effects with regards to which came first: asymptotic load lifting or the high-speed wind tunnel assay.

### Statistical analyses

All statistical analyses for Experiment 1 were performed using JMP Pro 14.2.0. To estimate repeatability for each type of flight performance assay, we calculated intraclass correlation coefficients (ICC) (Sokal and Rohlf, 1995) from the variance components partitioned for the random effect of individual in our generalized linear mixed models (GLMM, see below). To examine sexual dimorphism in *A. alexandri*, we tested for morphological differences between females and males using *t*-tests or the non-parametric Welch's *t-*test when the assumptions of normality and even variances were violated, as determined by Levene's test.

To test which variables affected top speed and mass lifted, we first ran a GLMM with mass lifted, sex, order in which the two assays were performed, year, trial number and calendar day on which the assays were performed as fixed effects, and individual ID as a random effect. Order, year, trial number and day were included as nuisance variables, to test for effects of tiring, learning or a seasonal decline in performance (i.e. if individuals performed better earlier in the summer). We dropped order, trial number and day because these terms were not significant but kept year as this was significant. For the final model, we used a single average value for top speed and mass lifted from each bird, and included year, sex and year×sex interaction effects. Including both sex and morphological variables in the GLM posed a multicollinearity problem, because there were pronounced sex differences for all three morphological variables. Therefore, we also ran a GLM on each sex separately and included the morphological parameters as factors.

### Experiment 2: between-species comparisons

Adult hummingbirds of all four species were captured in July, August and September 2019 and transported to UC Riverside. This time of year is not the breeding season and corresponds to the annual molt for *Calypte anna* (Lesson 1829), *Calypte costae* (Bourcier 1839) and *Selasphorus sasin* (Lesson, RP, 1829), although most individuals were not actively molting when captured. Both sexes of *S. sasin*, *C. anna* and *C. costae*, and males only of *A. alexandri* were sampled, resulting in seven species–sex categorizations. Each sampled bird was subjected to both the top speed assay and load lifting assay.

Maximum forward flight speed was measured as described above. The working section was the same, except for the replacement of deer netting (15 mm square) with mosquito netting (2 mm square) at the upstream end, because *C. costae* were small enough to fit through the deer netting. This difference in mesh produced a slightly different airspeed calibration from that in experiment 1, which we measured with a hot-wire anemometer (Amprobe TMA-21HW). We do not know whether/how the difference in mesh affected the turbulence intensity in the working section. Deer netting was also placed on the sides and top of the working section as a visual barrier, to reduce the tendency of the birds to try to escape by flying through the acrylic walls of the tunnel. Birds that repeatedly attempted to fly through the walls of the tunnel, or otherwise refused to fly, were dropped from the study (*N*=11) leaving 44 sampled birds. The protocol for these trials was modified slightly from that of experiment 1. After being placed in the working section, birds were acclimated to fly in the wind tunnel at 6 m s^−1^ for 2 min. After a 2 min rest, the next trial began and was repeated for a total of five top speed trials. As the birds tended to lose mass during the experiment (due to defecation), they were weighed again after completing all five trials. The two slowest trials were dropped for statistical analysis and the remaining three trials were averaged to determine the bird's mean top speed.

We assessed the wingbeat kinematics near a bird's top speed with high-speed videography. A high-speed camera (MIRO EX4, Vision Research, 500 or 1000 frames s^−1^) was placed ∼2 m behind (directly downstream) of the bird, outside the working section, to measure wingbeat kinematics (Movie 2). Video was analyzed from slightly before the bird's top speed (as defined above), because there was a tendency for kinematics to become erratic (gliding, bounding or fluctuating amplitude, with short bursts of high-amplitude flapping) when the bird was no longer able to maintain its forward position in the tunnel and began to drift back in the working section, right at its top speed. We measured wingbeat frequency and stroke amplitude from an average of 10 wingbeats. Stroke amplitude was digitized using the measure angle tool in ImageJ v1.53j (Schneider et al., 2012), assuming that the stroke plane was parallel to the image plane of the camera. We only used videos in which the bird was not visibly gliding or otherwise engaging in a maneuver (i.e. wingbeat kinematics were consistent across all 10 wingbeats).

### Wingbeat frequency at variable airspeeds

Hummingbird wingbeat frequency is relatively static across airspeeds (Tobalske et al., 2007) but has not yet been characterized at top speed. We recorded one bird from each measured species–sex class (except female *A. alexandri*) across a range of airspeeds to assess whether there was any change in wingbeat frequency as an individual approached its top speed. High-speed video was recorded at intervals of 1.6 m s^−1^ (starting at 0 m s^−1^) until the bird could no longer maintain its position in the working section. Wingbeat frequency measurements were taken after the focal bird had completed both the top speed and asymptotic load lifting assays.

### Asymptotic load lifting

The protocol for the load lifting trials followed that described in ‘General methods’, above, with a few modifications. We used a taller flight chamber (114×36.8×36.8 cm, to accommodate larger species that can lift more beads), increased the number of trials to five, and allowed each bird to rest for 2 min between trials. The lowest two trials were discarded, and the remaining three were averaged for analysis. As there were no order effects (all *P*>0.05, i.e. suggesting no evidence of fatigue) for the flight performance assays in experiment 1, the flight performance of all birds was assayed in the same order for experiment 2; first they were challenged with the top speed assay and then with the load lifting assay.

### Path analysis

Estimation of the individual effects of the parts of an integrated biological system like the flight apparatus is a major challenge. Advances in biomimetic flapping wing ‘ornithopters’ do not fully replicate the kinematics of an intact feathered bird in flight (Coleman and Benedict, 2025). However, morphological and kinematic traits are highly correlated within a species, e.g. larger individuals of a long-winged species will tend to have longer wings and larger flight muscles. Comparison among multiple species with different species-typical flight morphologies and degrees of sexual dimorphism is therefore necessary to isolate the effect of any one trait. By modeling the variance in morphological and kinematic traits explained by species identity and sex, we can estimate whether the remaining variance is still associated with differences in flight speed. This let us overcome the strong correlation between sex-linked morphology and flight phenotype encountered comparing male and female *A. alexandri* and instead allowed us to ask what effect a change in a trait has on flight speed more generally in hummingbirds.

We used path analysis (implemented in LAVAAN 0.6-6; Rosseel, 2012) in R v3.6.3 (https://www.r-project.org/) to disentangle the effect of changes morphological and kinematic variables on flight speed from species–sex class, which are highly correlated. In path analysis, hypothesized relationships between variables are specified by the modeler as a series of linear equations where some variables serve as mediators of others to better account for the observed distribution of covariances among variables (Graham, 2008). Path analysis is a useful method to determine whether an observed correlation between two variables remains important or significant after accounting for the covariance in a linking variable. For example, a smaller species might fly slower than a larger species, but the cause of slower flight is not species identity per se but rather the effect of shorter wing length on the bird's ability to produce thrust. In a path model, flight speed is then modeled with wing length as its direct cause, while species is the factor that causes variation in wing length; visually, each step of the model is depicted with a path from species to flight speed, passing through wing length. As a variable can be modeled as both being caused by one variable and causing another, variables are not really ‘dependent’ or ‘independent’ and are instead classified as exogenous or endogenous. Exogenous variables are not dependent on any other variable to explain their variance; that is, they are ‘causal’. Path analysis does not truly determine causality (no statistical test can do this); rather, we set up hypothesized causal/caused relationships using *a priori* assumptions and knowledge about the system and then compared the performance of each model. We classified species, sex and molt as exogenous variables; all others are endogenous. Endogenous variables have their own variance explained by at least one other variable and may also explain variance in another variable (Lleras, 2005). Assuming adequate biological justification for modeled paths, path analysis is a more realistic accounting of the correlated, interdependent nature of morphological and kinematic measurements.

**Fig. 2.**
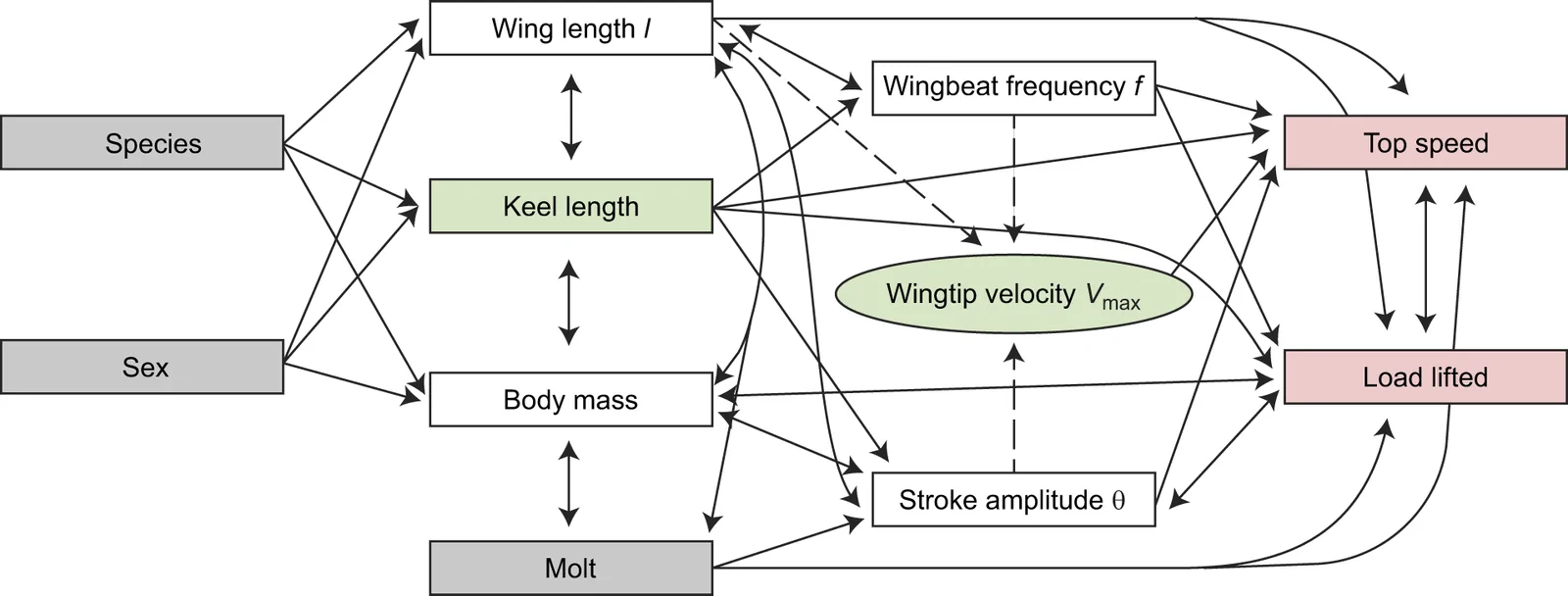
**Full path diagram illustrating all plausible relationships among our measured variables.** As the number of hypothesized relationships can be very large, equations in path models are visually represented as a path diagram. Empirically observed variables were directly measured and are depicted as rectangles, whereas the latent variable wingtip velocity (*V*_max_) was estimated from observed variables using Eqn 1 and is depicted as an ellipse. Hypothesized causal relationships are depicted as single-headed arrows pointing from cause to effect. A double-headed arrow between two variables represents a correlation term. Paths begin at endogenous variables, with individual paths traced by following one arrow to the next until reaching an endpoint (Wright, 1934). Gray indicates exogenous variables, white indicates endogenous variables, pink is the performance outcome and green indicates focal variables for kinematics and muscle size hypotheses. Note that keel length is also an endogenous variable. The dashed arrows are the paths associated with the latent variable (*V*_max_).

To construct our models, first we developed a ‘full’ model that represents all plausible relationships among our measured variables (Fig. 2). We considered relationships to be plausible if they made sense chronologically: species and sex are determined at fertilization, which subsequently determines morphological development, constraining kinematics and therefore performance limits. For example, sex is a plausible cause of wing length, but wing length cannot cause sex. As in all statistical models, it is problematic to go ‘fishing’ for a best-fit model, *post hoc*. We built one *a priori* path model for each hypothesis, muscle size and wing kinematics, and compared how well top speed was explained by each. LAVAAN 0.6-6 requires continuous, binary or ordinal categorical variables. To include ‘species’ as a nominal categorical variable in our path models, we used a leave-one-out approach with one species left out. This affects the interpretation of the paths associated with ‘species’ (wing length, body mass, keel length) to be the comparison of all species relative to the one left out (Rosseel, 2012). The species chosen to be ‘left out’ does not qualitatively change the results and the models are robust to which species is left out. We chose *C. anna* to be left out because it is the largest species.

In the muscle size model (*m*), top speed is primarily limited by available flight muscle power. Power output scales allometrically with body size in hummingbirds (Chai and Dudley, 1999). The keel is the site of attachment for the two primary flight muscles, the pectoralis and supracoracoideus, and the total mass of the pectoralis in an individual is strongly correlated with keel length in hummingbirds (Wright and Steadman, 2012), so we measured keel length as a proxy for overall flight muscle size (Zusi, 2013). We did not allow any paths from kinematic variables (stroke amplitude, wingbeat frequency, wingtip velocity) to flight speed in the muscle size model.

In the kinematics model (*k*), wingtip velocity (*V*_max_) limits top speed. Wingtip velocity is represented as a latent variable (∼*V*_max_) in the model because it was estimated from other measured variables. Assuming the wingtip oscillates under simple harmonic motion following Clark and Mistick (2018), ∼*V*_max_ of the wingtip is simply:

(1) $$∼V_{\max}=\pi l\theta f,$$

where *l* is wing length, θ is stroke amplitude, *f* is wingbeat frequency and π is a constant. Thus, the latent variable ∼*V*_max_ is a product of three endogenous variables: wing length, wingbeat frequency and stroke amplitude. We used high-speed video to estimate stroke amplitude and wingbeat frequency from 10 steady-state wingbeats of hummingbirds flying near (but not necessarily precisely at) their top speed. We did not allow any paths from keel length to flight speed in the kinematics model.

To test the hypothesis that load lifting and top speed are correlated metrics of flight performance, we included a correlation term between top speed and load lifting in both models. Load lifting performance is hypothesized to be constrained by the same factors that affect top speed: wing kinematics and flight muscle output (Chai et al., 1997). However, to prevent indirect effects of kinematics variables in the muscle model and vice versa, we constrained load lifting to follow the same rules as top speed in each model (Fig. 5): in the kinematics model, load lifting cannot be associated with muscle size (but kinematic variables are allowed), while kinematic variables are not allowed to influence load lifting in the muscle model (see Results, ‘Path modeling’). In both models, load lifting is correlated with body mass (Altshuler et al., 2010). In the kinematics model, load lifting has paths from wingbeat frequency and stroke amplitude (Chai et al., 1997). We included wing length as a correlation term because it is our closest measurement to wing area, which strongly influences how muscle force is translated into lift during takeoff (Norberg, 1995; Stiles et al., 2005). In the muscle size model, load lifting has paths from keel and wing length. If some birds are better overall at flying, then top speed and load lifting will be positively correlated in both models. Conversely, there may be a tradeoff between flight speed and load lifting ability, which would be reflected as a significant negative correlation in both models. If the correlation term is not significant in both models, then the contribution of kinematic and muscle traits is likely different for each trait (e.g. muscle size is more important in load lifting, while kinematics are more important in top speed).

Finally, we built one additional model *post hoc* based on the results of our *a priori* models:, the combined model (*c*). The combined model includes paths from both kinematic variables and keel length on both top speed and load lifting ability to assess the effects of disentangling muscle size and wingtip velocity from one another. Speed and load lifting are together caused by wingtip velocity and keel length. All other paths within this *post hoc* model were chosen based on the *a priori* model results, discussed below.

### Ethics

Techniques were approved by UC Riverside animal care and use committees (IACUC; protocols #20130018, #20160039 and #20190019). Birds were captured under permits from the US Fish and Wildlife Service (#MB087454-0), US Geological Survey Bird Banding Lab Permit (#23516) and California Department of Fish and Wildlife permit (#006598).

## RESULTS

### Experiment 1: sexual dimorphism in black-chinned hummingbirds

Morphometrics for each age–sex category of bird are given in Table 2, along with maximum speed and mass lifted.

**Table 2.** Morphometrics and performance traits by species–sex category

Species	Sex	*N*	Exp.	Mass (g)	Wing length (mm)	Keel length (mm)	WBF (Hz)	θ (deg)	∼*V*_max_ (m s^−1^)	Speed (m s^−1^)	Mass lifted (g)	Body load
*C. anna*	♂	6	2	4.52±0.36	48.19±1.47	19.86±0.23	39.58±1.97	153.5±6.82	18.04±0.74	16.24±0.95	6.04±0.77	1.53±0.16
	♀	10	2	4.01±0.29	48.36±1.73	17.94±0.39	37.4±2.09	137.02±7.07	15.7±1.34	15.6±1.15	5.18±0.83	1.43±0.25
*S. sasin*	♂	5	2	3.46±0.34	37.74±0.97	17.77±0.3	54.69±2.49	157.33±3.71	19.76±0.87	16.2±1.07	3.25±0.59	1.13±0.27
	♀	6	2	3.55±0.15	41.92±1.14	17.55±0.65	46.66±2.57	153.22±9.92	18.4±2.01	15.77±0.67	4.42±0.5	1.38±0.19
*C. costae*	♂	7	2	3.15±0.14	43.27±1.5	16.97±0.28	41.46±1.27	141.97±8.35	16.82±1.19	15.01±0.89	4.7±0.88	1.65±0.25
	♀	6	2	3.03±0.17	44.62±1.72	16.3±0.51	36.42±1.18	132.16±4.64	14.19±0.79	14.22±0.68	4.49±0.48	1.65±0.23
*A. alexandri*	♂	4	2	3.24±0.17	41.88±1.56	17.03±0.53	48.59±2.65	136.34±3.28	17.8±1.99	14.59±0.85	3.11±0.33	1.13±0.17
	♂	31	1	3.09±0.32	40.5±0.85	16.26±0.58	–	–	–	15.96±1.03*	3.29±0.42	1.03±0.19
	♀	22	1	3.42±0.21	45.0±1.4	15.6±0.52	–	–	–	15.1±1.1*	3.55±0.53	1.04±0.17

*N* is the number of birds. WBF, wingbeat frequency near top speed; θ, stroke amplitude near top speed; ∼*V*_max_, estimated maximum wingtip velocity at top speed (relative to the bird). Mass lifted is only the mass of the beads lifted. Body load is the ratio of load lifted to body mass. We report body load to facilitate comparison with similar studies. *The airspeed calibration for the wind tunnel differed between experiments 1 and 2.

#### Top speed

**Fig. 3.**
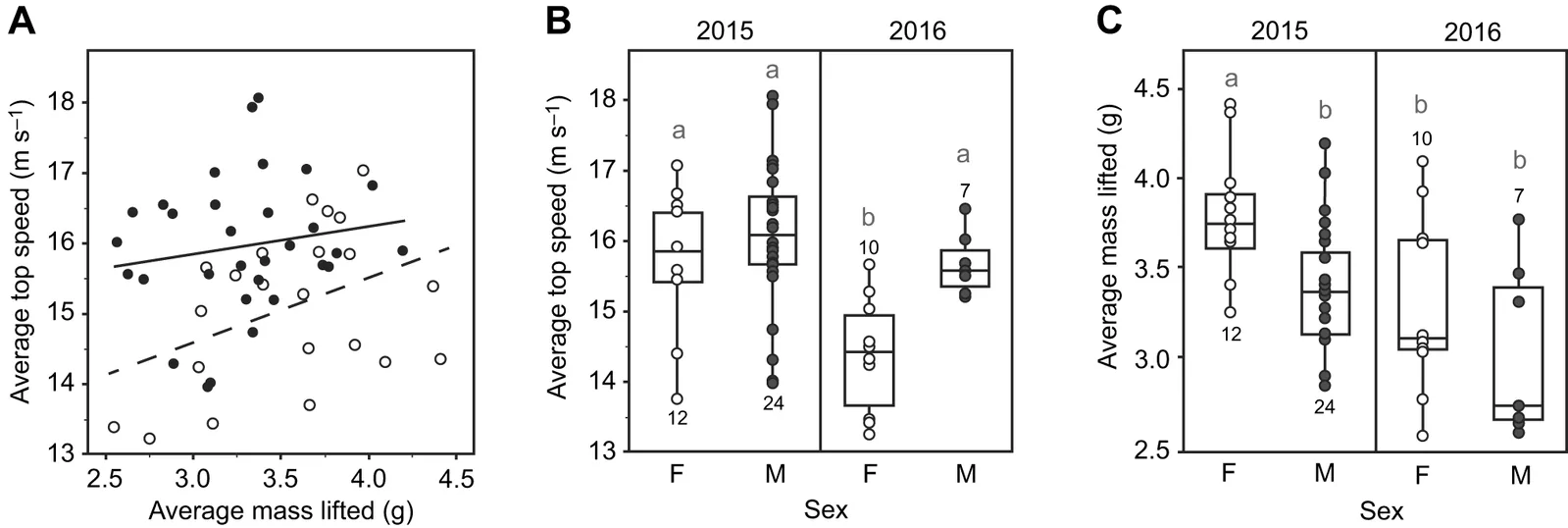
**Flight performance of female (*N*=22) and male (*N*=31) black-chinned hummingbirds (*Archilochus alexandri*) (experiment 1).** (A) There was a positive relationship between top speed flight and mass lifted (GLMM, *t*=2.41, d.f.=148.23, *P=*0.017), which is driven primarily by females (open circles; GLMM, *t*=1.81, d.f.=56.64, *P=*0.075) as the relationship was not significant for males (filled circles; *P=*0.11). (B) The difference in top speed flight performance between females and males varied by year; males had higher top speeds in the wind tunnel (GLMM, *t*=−2.89, d.f.=53.29, *P=*0.0056). There was a significant year effect: females (*t*=3.71, d.f.=18.42, *P=*0.0016), but not males (*P=*0.47), flew faster in 2015 relative to 2016. (C) Overall, females lifted more mass than males (*t*=3.73, d.f.=51.50, *P=*0.0005). Birds lifted more mass during the asymptotic load lifting assay in 2015 relative to 2016 (*t*=2.64, d.f.=52.95, *P=*0.011). This effect was not evident in females *(P=*0.36) but was marginally non-significant in males (*t*=1.89, d.f.=26.30, *P=*0.069). For box and whisker plots (B and C), the horizontal line is the median; upper and lower borders of the box and whiskers are quantiles and maximum–minimum, values, respectively. Numbers indicate sample sizes (*N*) for each sampled group. Letters indicate significant differences among group means following *post hoc* Tukey tests.

We tested 53 adult *A. alexandri* (22 females, 31 males). Repeatability was high in both the top speed assay (all ICC=0.83; females ICC=0.84; males ICC=0.82). In the initial models, year had a significant effect (final GLMM, *t*=2.38, d.f.=54.13, *P*=0.021), but the other nuisance variables (trial number, performance assay order and date) had no effect (all *P*>0.16) and were dropped. In the final model, year had a significant effect (Fig. 3B,C). Males flew faster (mean±s.d.: males 16.0±1.0 m s^−1^; females 15.1±1.1 m s^−1^) in the wind tunnel (Fig. 3B), but mass lifted was not correlated with top speed (Fig. 3A).

In GLMMs considering each sex separately, there was a positive association between top speed and mass lifted in females, but this relationship was not significant (*t*=1.81, d.f.=56.64, *P*=0.0749). No relationships were detected between top speed and any morphological measure in females (keel length: *P*=0.18; wing length: *P*=0.11, body mass: *P*=0.15), but there was a significant year effect. In males, top speed and mass lifted were not significantly associated (Fig. 3A; *P*=0.11), and there was no significant relationship between top speed and morphology (keel length: *P*=0.82; wing length: *P*=0.33 or body mass: *P*=0.28), nor was there a year effect (*P*=0.47).

#### Load lifting

As load lifting ability was not a significant predictor of top speed in *A. alexandri*, we investigated its correlates separately in another model. Repeatability was high for this assay (all ICC=0.75; females ICC=0.73; males ICC=0.80). Because they were not significant, flight assay order, trial number and date were not included in the final model (all *P*>0.25). Sex and year had significant effects on mass lifted (Fig. 3C).

Considering sexes in separate GLMMs, morphological measures and year were not significant on load lifting ability for females (*P*>0.26) and top speed was marginally non-significant (*t*=1.74, d.f.=55.24, *P*=0.088). In males, none of the morphological variables (body mass, wing length and keel length *P*>0.26) or top speed was significant (*P=*0.11). Only the year effect on load lifting ability approached significance (Fig. 3C; *t*=1.89, d.f.=26.30, *P=*0.069).

### Experiment 2: between-species comparisons

**Fig. 4.**
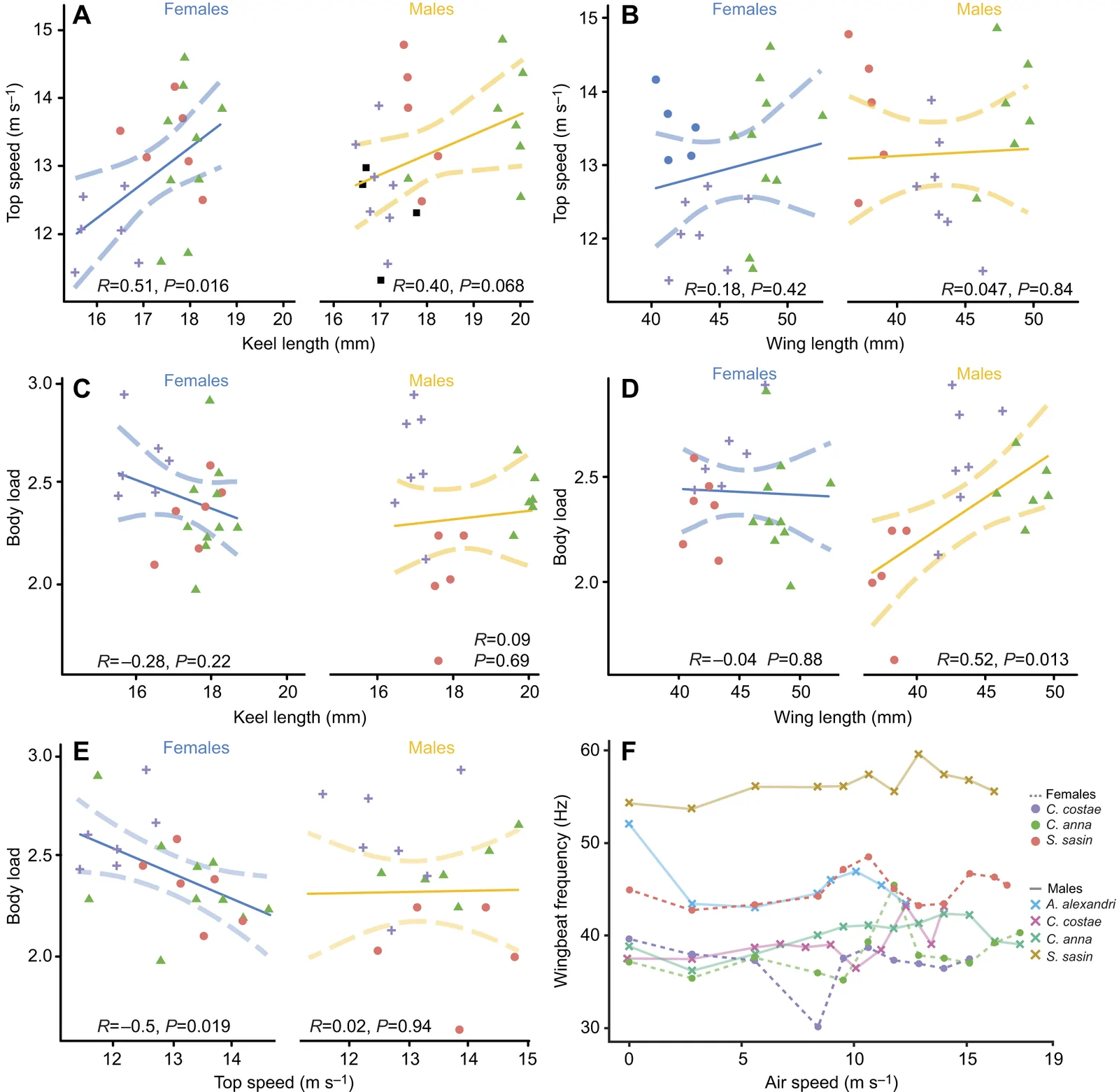
**Raw flight performance prior to decomposition with path analysis in four species of male and female hummingbirds (experiment 2).** (A–D) Simple linear regression by sex for keel and wing length against top speed (A,B) and body load (C,D) (a unitless ratio of total mass lifted to body mass) in male (*N*=23) and female (*N*=21) hummingbirds. We report body load to facilitate comparison with other studies. Dashed line indicates standard error. Top speed is positively associated with (A) keel length in females (*R*=0.51, *P*=0.016) and males (*R*=0.40, *P*=0.068) but not with (B) wing length (females *P*=0.42, males *P*=0.84). Body load lifted is not significantly correlated with (C) keel length in either sex (females *P*=0.22, males *P*=0.69) or with (D) wing length in females (*P*=0.88) but is significantly correlated with wing length in males (*R*=−0.52, *P*=0.013). (E) Body load is negatively correlated with top speed in females (*R*=−0.5, *P*=0.019) but not males (*P*=0.94), as in experiment 1. (F) Wingbeat frequency by airspeed in one of each species–sex class (except female *A. alexandri*). Wingbeat frequency changes little across airspeed.

We measured a total of 44 adult hummingbirds (22 females, 22 males), with *N*=4–10 for the seven species–sex categories (we caught no female *A. alexandri*). Six individuals were actively molting primary flight feathers: 3 female *C. anna*, 2 female *C. costae* and 1 male *C. costae*. Repeatability was high for both top speed (ICC=0.93) and load lifting assays (ICC=0.90). Linear regressions of speed, body load (body mass plus mass lifted divided by body mass), keel length and wing length are summarized in Fig. 4. There was a pronounced sex difference in the relationship between top speed and load lifting ability. Top speed was significantly associated with keel length (Fig. 4A) but not wing length in both males and females (Fig. 4B). Keel length was significantly associated with body load lifted in females but not males (Fig. 4C), while wing length was significantly correlated with body load lifted in males but not females (Fig. 4D). While there was no correlation between top speed and load lifting ability in males (*R*^2^=0.02, *P*=0.94), there was a significant negative correlation in females (*R*^2^=−0.50, *P*=0.019) (Fig. 4E).

Wingbeat frequency over varying flight speeds from *N*=7 birds (*N*=1 of each of the species–sex categories) is shown in Fig. 4F. Wingbeat frequency did not vary with airspeed (Fig. 4F). Males overall had higher wingbeat frequencies than females (Table 2).

#### Path modeling

**Fig. 5.**
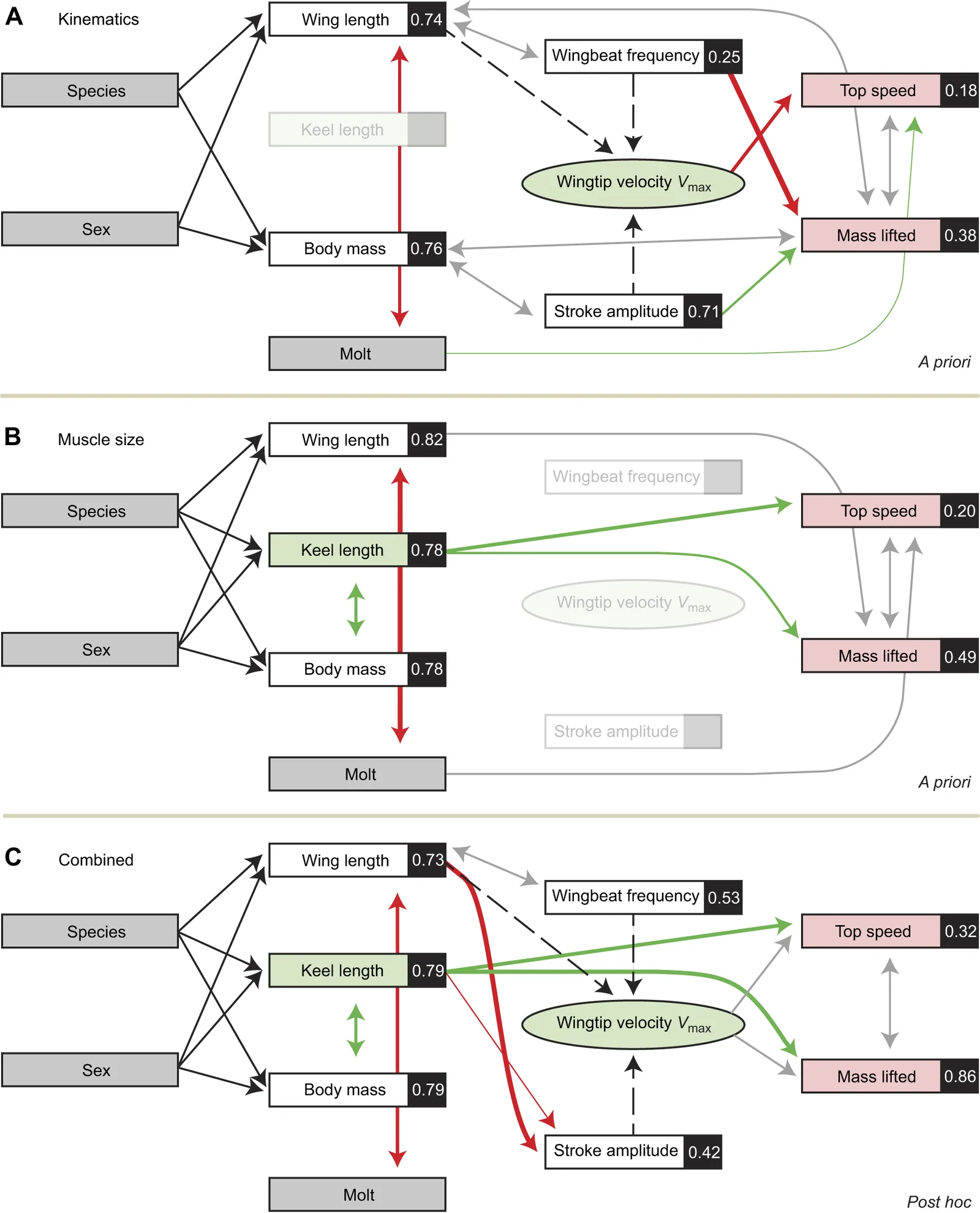
**Path models.** (A) Kinematics, (B) muscle size and (C) combined models. Each model is a subset of the full model (illustrated in Fig. 2). Green arrows indicate significant, positive β association between variables. Red arrows indicate significant, negative β association. Black arrows indicate associations between nominal variables (e.g. species). Gray arrows indicate non-significant associations. Dashed arrows lead to the latent variable. Arrow widths are scaled to their standardized β estimate to facilitate comparison between the models. Each variable's *R*^2^ is indicated next to the trait. For simplicity, no coefficients are indicated for ‘species’, as the model was run with a leave-one-out approach and thus represents three species, each compared against the left-out species (*C. anna*); see Materials and Methods for more details. For ‘sex’, females were coded as 0 and males as 1, so sex is best interpreted as ‘effect of being male’. All model statistics are given in Tables S1–S3.

The models used for path analysis, correlation coefficients, fit statistics and results are described in Table 3 and illustrated in Fig. 5. We report standardized β estimates here to facilitate comparison between the models. All model statistics, including unstandardized solutions, are available in Tables S1–S3. Unstandardized solutions are useful to infer how much an increase in one unit of one factor changes the outcome of another factor (e.g. 1 mm increase in keel length increases top speed by ∼0.4 m s^−1^ in the muscle model). The data and scripts used to reproduce the models can be found in Dataset 1 and Script 1.

**Table 3.** Summary statistics for path models

Model	CFI	RMSEA	SRMR	AIC	*R* ^2^						
					Wing length	θ	WBF	Body mass	Keel length	Mass lifted	Top speed
Kinematics	0.584	0.323	0.225	1039.09	0.743	0.706	0.252	0.762	–	0.380	0.183
Muscle size	0.908	0.178	0.102	522.28	0.822	–	–	0.783	0.784	0.491	0.204
Combined	0.732	0.264	0.182	1080.02	0.734	0.422	0.531	0.793	0.785	0.855	0.321

AIC, Akaike information criterion; CFI, comparative fit index; RMSEA, root mean square error of approximation; SRMR, standardized root mean square residual. Excluded variables are indicated by dashes.

#### Top speed

In the kinematics model (*k*), top speed was significantly negatively associated with the main causal factor ∼*V*_max_ (β*_k_*=−0.427, *P_k_*=0.038). Wing length was strongly associated with species but not with sex (*P_k_*=0.1). Of the correlation terms, only the negative correlation between wing length and molt status remained significant (*P_k_*=0.011, β*_k_*=−0.388); all other correlations were non-significant (Table S1). Body mass was strongly associated with species and sex, as expected (all *P_k_*<0.01; Table S1). The kinematics model overall explained low amounts of variance in top speed (*R*^2^*_k_=*0.18).

In the muscle size model (*m*), top speed was significantly associated with keel length (*P_m_*=0.001). Faster speeds were associated with longer keels (β*_m_*=0.451). Body mass, wing length and keel length were all significantly associated with species and sex, as expected (Table S2). Body mass retained a relatively large effect on keel length (β*_m_*=0.387). The muscle model had marginally higher explanatory power for top speed (*R*^2^*_m_=*0.20). The fit statistics of the muscle model were substantially better than the kinematics model (Table 3).

#### Load lifting

Load lifting was modeled alongside top speed in both the kinematics and muscle size path models. In the kinematics model, wingbeat frequency (*P_k_*<0.001) and stroke amplitude (*P_k_=*0.022) were significant predictors of load lifting ability, with moderate explanatory power (*R*^2^*_k_=*0.38). Differences in wingbeat frequency (β*_k_*=−0.680) had a stronger effect on lifting performance than differences in stroke amplitude (β*_k_*=0.306). In the muscle model, keel length (*P_m_*=0.007) and wing length (*P_m_*<0.001) were both significantly associated with load lifting performance (Table S2). The correlation term between load lifting and top speed was not significant in either model (*P_k_*=0.14, *P_m_*=0.41).

#### *Post hoc* combined model

As top speed is poorly explained by our *a priori* models, we constructed a third model to evaluate the combined effects of wing kinematics and muscle size on top speed (Fig. 5C; Table S3). In the combined model (*c*), both top speed and load lifting performance were significantly correlated with keel length (*P_c_=*0.001) but not ∼*V*_max_ (*P_c_=*0.36). Despite this, the correlation term between load lifting and top speed was not significant (*P_c_=*0.704). Unsurprisingly, this model explained more variance in top speed (*R*^2^*_c_=*0.32) and load lifting (*R*^2^*_c_=*0.85) than either *a priori* model (Table 3). The effect size of keel length on top speed was the same in both the combined and muscle size models (β*_c_*=0.452 versus β*_m_*=0.451). However, the effect of keel length on load lifting was greater in the combined model (β*_c_*=0.538 versus β*_m_*=0.305).

## DISCUSSION

### Correlates of fast flight in hummingbirds

To fly fast, a hummingbird must produce both lift and thrust. We hypothesized that an individual's maximum forward flight speed is primarily thrust limited, as lift generated from air flowing over the body and wings increases substantially at higher airspeeds. Thrust is the forward-directed force from the motor flapping the wings minus the drag force. We investigated morphological and kinematic correlates of flight speed in hummingbirds to test the hypothesis that thrust is primarily limited by either motor power or wingbeat kinematics. Consistent with previous findings within a single species and with expectations from their behavioral ecology, males (which tend to have shorter wings and longer keels) flew faster than females. Using a series of path models to disentangle species and sex from morphology and kinematics, we identified keel length (our proxy for muscle size) as the most significant predictor of flight speed. Flight kinematics, which are well known to be correlated with maximum power output as measured by a load lifting assay, were not significantly associated with flight speed in the best-fitting model. This, combined with the lack of any relationship between load lifting ability and top speed, suggests forward flight speed and stationary hovering performance are not directly comparable measures of a common ‘performance’ ability.

Keel length was the most significant predictor of top speed in the combined model, which was the main prediction of the muscle size hypothesis. In both the muscle size (Table S2) and combined models (Table S3), a 1 mm longer keel was associated with ∼0.37 m s^−1^ gain in top speed, a considerable increase. This effect remained despite accounting for variance in keel length caused by species, sex and body mass. Keel length is significantly correlated with stroke amplitude (Pearson's *r*=0.3, *P*=0.044) but not wingbeat frequency (Pearson's *r*=−0.014, *P*=0.93), which aligns with observations that wingbeat frequency in hummingbirds is constrained by neuromuscular physiology (Mahalingam and Welch, 2013).

Keel length is easy to measure on a living bird but is a comparatively coarse estimate of flight muscle mass, reducing model fit. Pectoral muscle size can and does vary substantially among individuals (Lindström et al., 2000) and throughout the annual cycle, shrinking during molt (Chai et al., 1997) and increasing in size during the breeding season (N.A.N., unpublished). These dynamics may explain the significant effect of year in experiment 1 (Fig. 3) as we measured *A. alexandri* towards the end of their breeding season. The summers of 2015 and 2016 when these birds were sampled for experiment 1 were also the last years of an extreme drought in California (Prugh et al., 2018). Birds captured in 2016 may therefore have been in worse physical condition (i.e. underweight relative to their normal, healthy mass) compared with those captured in 2015. Given these sources of variability, we suggest future experiments measuring top speed should measure muscle size directly and not use a skeletal correlate as we did here.

If greater muscle power permits an increase in stroke amplitude while maintaining constant wingbeat frequency, we would expect wingtip velocity to be positively associated with top speed, which we did not observe. Wingtip velocity was significantly negatively associated with but explained little variation in top speed only in the kinematics model, and the significant association vanished in the combined model (Table 3) despite highly repeatable top speed measurements (ICC values ranged between 0.82 and 0.93). One cause of this pattern might be the relatively erratic nature of flight at top speed. In the Introduction, we implied that top speed is a simple balance between thrust and drag (Fig. 1). However, our qualitative observations of bird behavior during the assay suggest a role for transient burst performance. Some individuals would cast from side to side or repeatedly accelerate forward in bursts once they began to struggle to maintain their position. The wingbeat kinematics we recorded in experiment 2 were simple (stroke amplitude and wingbeat frequency averaged over 10 wingbeats), but we could not reliably record these simple kinematics at the most relevant moment of the assay, just after a bird has hit its top speed, because these kinematics become increasingly erratic.

As part of our wing kinematics hypothesis, we suggested birds increase their stroke amplitude and/or wingbeat frequency at top speed to protect the advance ratio. Wingbeat frequency is highly consistent across airspeeds (Fig. 4F), and wing length is constant, so stroke amplitude remains the single easiest target to assess kinematic transience in our data. We observed six hummingbirds that had at least one or two unsteady wingbeats recorded at stroke amplitudes exceeding 170 deg: two female *S. sasin*, one male *S. sasin* (Movie 2), one male and one female *C. costae*, and one male *C. anna*. Many other individuals increased their stroke amplitude transiently 10–20 deg above their average during top speed, but most birds were well below maximum observed amplitudes (Table S2). This transience was not captured in either our stroke amplitude metric or the wingtip velocity latent variable in our path models. While hummingbird flight at low airspeeds is kinematically more similar to that of insects than to other birds (Song et al., 2016), and stability is a major limiting factor in insects, such as orchid bees (Combes and Dudley, 2009), we do not think problems maintaining stability were the cause of erratic kinematics. The birds in our study rarely crashed, and when they did it was because they hit an obstacle (usually the side of the wind tunnel). Perhaps the greater coupling between body angle and stroke plane angle in insects makes them more prone to instability than birds. High-speed videography of side and rear views taken simultaneously over a longer time course would help resolve the evolution of flight kinematics as birds approach and reach their top speed.

### Top speed is not associated with load lifting ability

The load lifting assay we used has been employed by many previous studies as a measure of hummingbird flight performance (e.g. Altshuler et al., 2010; Altshuler and Dudley, 2002, 2003; Chai et al., 1997; Chai and Millard, 1997; Groom et al., 2017; Mahalingam and Welch, 2013). As a measure of the maximal power of the flight apparatus, we hypothesized that load lifting performance would be correlated with top speed if muscle power, but not wingbeat kinematics, is the main limit to faster flight. While keel length was a significant predictor of flight speed, consistent with the muscle size hypothesis, load lifting was not significantly associated with top speed in any path model. This may constitute support for the kinematics hypothesis, where drag forces are so high at top speed that spending reserve power from the flight apparatus does not translate to higher flight speeds. Instead, birds reach their top speed at some optimal kinematic combination for their wing length. During maximal loading, hummingbirds typically achieve stroke amplitudes that approach a geometric limit of 180 deg at constant wingbeat frequency (Chai et al., 1997) but rarely exceeded 160 deg during fast forward flight (this paper). Keel length is a significant predictor of stroke amplitude, but total body mass is not (Table S3). Altogether, these results indicate muscle power is indeed the primary limitation on stroke amplitude and forward flight speed, but birds do not use all available power during fast flight.

Hummingbirds were sexually dimorphic in all measured traits and flight performance assays in both experiments 1 and 2. Generally, males were faster than females, while females lifted as much or more than males (Table 2). While all hummingbirds hover to forage, only females provide parental care and must spend much more of their flight time hovering to catch tiny flying insects and collecting nectar (Clark, 2022), the predominant foods they feed their chicks (Carpenter and Castronova, 1980; Remsen et al, 1986; Yanega, 2007). Males only forage enough to feed themselves and instead spend much more of their time in fast flight. Males defend widely spaced courtship territories which they routinely leave to harass and chase neighboring males, particularly during the breeding season. These high-speed chases can last for several minutes, with the antagonists flying in large circles back and forth in the airspace over their territories (Clark and Mitchell, 2020). Males also pursue females to perform courtship displays such as high-speed dives (Clark, 2009), and if a male overtakes a female, he may force her to land or take cover. While females also aggressively defend small territories around their nests, these chases tend to be shorter, and females do not perform flight displays. There is thus an ecological basis for sex-specific differences in flight performance optima (Stiles et al., 2005).

Sex-specific flight optima caused by variation in flight muscle composition could also explain the apparent lack of association between top speed and load lifting ability. The pectoralis major can be up to 10 times larger than the supracoracoideus in other bird groups but is only about twice as large in hummingbirds (Deeming, 2023; Greenewalt, 1962). The relatively large supracoracoideus generates substantial lift on the upstroke, permitting stationary flight (Warrick et al., 2012). During fast forward flight, hummingbird wingbeat kinematics become more bird-like with a greater emphasis on the downstroke (Fig. 1B). Selection for fast flight in males may therefore drive investment in a larger pectoralis (relative to females) and shorter wings to maximize thrust, while selection for efficient hovering in females may promote investment in a large supracoracoideus and longer wings to maximize lift. Our analysis only included a proxy of total mass, which masks any muscle-specific effects. Any investigation of a relationship between flight speed and hovering ability may need to account for the relative composition of the flight muscles and not only their absolute mass.

The lack of a consistent positive relationship between load lifting ability and top speed, both within individuals and between species, suggests burst capacity measured as load lifting and top speed cannot be used as proxy measurements of a generalized flight ‘performance’. Despite high repeatability for both flight assays, the combined model only explained about 32% of the variance in flight speed compared with 85% of the variance in load lifting ability (Table 3). What determines variation in flight speed remains to be explored, but the missing variables are unlikely to be identified by focusing on burst capacity. Flight is the product of coordination among elements of the flight apparatus and their contributions should be considered separately. Just as differences in the relative sizes and composition of muscles and limbs in terrestrial vertebrates result in various capacities for endurance, speed and strength, changes in each element of the flight apparatus do not have directional effects on flight in aerial vertebrates.

Most previous studies of hummingbird flight only sampled one sex, often only adult males (e.g. Altshuler et al., 2012), or sometimes only females (e.g. Tobalske et al., 2007), usually from limitations on sampling during the breeding season or from difficulties in identification. While the most common bird models of flight performance (e.g. hummingbirds, pigeons, owls) vary greatly in the extent and type of their behavioral and morphological sexual dimorphism, dimorphism is not usually explicitly considered or discussed as a source of flight variation. In manakins, the flight muscles are sexually dimorphic and have androgen receptors (Schlinger et al., 2013), and hence male muscle physiology responds to annual changes in testosterone. Whether hummingbirds also have sexually dimorphic responses to a sex hormone and variation in their annual flight performance would be of interest for future study. This example highlights a pattern in flight performance literature, which is to pick one age/sex class (usually adult males) and use them to represent all members of the species. The work laid out here has, we hope, made clear why this approach will underestimate or miss important explanatory variation in flight performance. Many groups use hummingbirds for ‘bioinspiration’ to design hovering robots; females may be better models for hovering than males, as females outperform males at load lifting while males outperform females at high-speed flight (Figs 3 and 4).

## Supplementary Material

10.1242/jexbio.251012_sup1 Supplementary information

Dataset 1. Spreadsheet with raw data inputs for Experiment 1 (GLM) and Experiment 2 (path modeling).
