# Supplementary material for: Effects of wingbeat kinematics and muscle size on maximum flight speed in hummingbirds
Source: J Exp Biol. 2026 Aug 21;229(16):jeb251012. doi: 10.1242/jeb.251012 (PMC13539427; doi:10.1242/jeb.251012)
Supplement: Supplementary file 1 [file jexbio-229-251012-s1.pdf]

Table S1. Summary of regression, covariance, and variance results for the kinematics path model. Bold P-values are significant at < 0.05. **Estimate** is the unstandardized solution, given as units of the dependent variable (e.g., 1mm increase in keel length increases stroke amplitude by 5.5º). **Std All** is the standardized solution, given as a unitless coefficient of correlation to facilitate comparison within and between models. **WBF** = wingbeat frequency, tip velocity = ~V_{max} (from Equation 1). Species regressions are leave-one-out with ANHU left out, so path estimates including species as a term should be interpreted as 'relative to ANHU'.

Wingbeat Kinematics Model

	Variable	Estimate	Std Err	Z	P	Std All
Regressions:	speed					
	~ tip velocity	-0.402	0.194	-2.070	**0.038**	-0.427
	mass lifted					
	~ WBF	-0.130	0.025	-5.306	**< 0.001**	-0.680
	~ stroke amplitude	0.031	0.013	2.293	**0.022**	0.306
	wing					
	~ sex	-0.736	0.455	-1.633	0.102	-0.113
	~ COHU	-4.349	0.524	-8.308	**< 0.001**	-0.606
	~ ALHU	-6.011	0.550	-10.94	**< 0.001**	-0.790
	~ BCHU	-4.863	0.812	-5.987	**< 0.001**	-0.440
	mass					
	~ sex	0.203	0.080	2.524	**0.012**	0.190
	~ COHU	-1.100	0.093	-11.79	**< 0.001**	-0.936
	~ ALHU	-0.651	0.098	-6.652	**< 0.001**	-0.522
	~ BCHU	-1.001	0.145	-6.919	**< 0.001**	-0.553
Covariances:	.speed					
	~~ .load lifting	-0.185	0.126	1.468	0.142	0.237
	~~ molt	0.036	0.044	0.832	**0.036**	0.120
	.wing					
	~~ .WBF	-3.676	2.128	-1.728	0.084	-0.429
	~~ molt	-0.225	0.088	-2.542	**0.011**	-0.388
Variances:	mass lifted					
	~~ mass	0.065	0.037	1.752	0.080	0.276
	~~ wing	0.169	0.184	0.921	0.357	0.113
	stroke amplitude					
	~~ mass	0.365	0.405	0.902	0.367	0.225
	.speed	0.739	0.183	4.042	**< 0.001**	0.817
	.load lifting	0.828	0.180	4.592	**< 0.001**	0.620
	.wing	2.699	0.801	3.370	**0.001**	0.257
	.BMF	0.067	0.015	4.592	**< 0.001**	0.238
	.stroke amplitude	39.19	49.71	3.370	**< 0.001**	0.257
	.WBF	27.26	7.816	3.488	**< 0.001**	0.748
	molt	0.124	0.027	4.590	**< 0.001**	1
	tip velocity	1.020	0.760	1.341	0.180	1

Journal of Experimental Biology • Supplementary information

Table S2. Summary statistics for muscle size path model.

Muscle Size Model

	Variable	Estimate	Std Err	Z	P	Std All
Regressions:	speed					
	~ keel	0.373	0.111	3.358	**0.001**	0.451
	mass lifted					
	~ keel	0.315	0.116	2.712	**0.007**	0.305
	~ wing	0.158	0.032	4.897	0.158	0.545
	wing					
	~ sex	-1.921	0.469	-4.090	**<0.001**	-0.241
	~ COHU	-4.264	0.552	-7.732	**< 0.001**	-0.488
	~ ALHU	-8.591	0.574	-14.97	**< 0.001**	-0.933
	~ BCHU	-5.653	0.869	-6.508	**< 0.001**	-0.407
	mass					
	~ sex	0.211	0.081	2.598	**0.009**	0.191
	~ COHU	-1.173	0.096	-12.22	**< 0.001**	-0.973
	~ ALHU	-0.707	0.100	-7.082	**< 0.001**	-0.557
	~ BCHU	-1.039	0.150	-6.926	**< 0.001**	-0.543
	keel					
	~ sex	1.075	0.168	6.409	**< 0.001**	0.478
	~ COHU	-2.244	0.197	-11.39	**< 0.001**	-0.910
	~ ALHU	-1.096	0.205	-5.344	**< 0.001**	-0.422
	~ BCHU	-2.300	0.310	-7.410	**< 0.001**	-0.588
Covariances:	.speed					
	~~ .load lifting	-0.085	0.104	-0.820	0.412	-0.125
	~~ molt	0.010	0.037	0.263	0.792	0.034
	.wing					
	~~ molt	-0.287	0.097	-2.948	**0.003**	-0.496
	.keel					
	~~ .mass	0.052	0.021	2.428	**0.015**	0.387
Variances:						
	.speed	0.688	0.147	4.691	**< 0.001**	0.796
	.load lifting	0.683	0.146	4.690	**< 0.001**	0.509
	.wing	2.837	0.605	4.690	**< 0.001**	0.178
	.BMF	0.066	0.014	4.713	**< 0.001**	0.217
	molt	0.118	0.025	4.692	**< 0.001**	1.000
	.keel	0.273	0.058	4.690	**< 0.001**	0.216

Journal of Experimental Biology • Supplementary information

Table S3. Summary statistics for the combined path model.

Combined Model

	Variable	Estimate	Std Err	Z	P	Std All
Regressions	speed					
	~ wingtip velocity	-0.487	0.528	-0.923	0.356	-0.341
	~ keel	0.373	0.109	3.420	**0.001**	0.452
	mass lifted					
	~ wingtip velocity	1.308	1.262	1.036	0.300	0.752
	~ keel	0.541	0.099	5.451	**<0.001**	0.538
	wing					
	~ sex	-1.062	0.473	-2.243	**0.025**	-0.158
	~ COHU	-4.257	0.550	-7.740	**<0.001**	-0.158
	~ ALHU	-6.650	0.577	-11.52	**<0.001**	-0.845
	~ BCHU	-4.628	0.853	-5.423	**<0.001**	-0.405
	mass					
	~ sex	0.241	0.082	2.922	**0.003**	0.217
	~ COHU	-1.160	0.096	-12.04	**<0.001**	-0.944
	~ ALHU	-0.674	0.100	-6.713	**<0.001**	-0.518
	~ BCHU	-1.130	0.149	-7.597	**<0.001**	-05.98
	keel					
	~ sex	1.114	0.175	6.363	**<0.001**	0.487
	~ COHU	-2.223	0.203	-10.93	**<0.001**	-0.897
	~ ALHU	-1.063	0.213	-4.981	**<0.001**	-0.396
	~ BCHU	-2.324	0.316	-7.365	**<0.001**	-0.597
	stroke amplitude					
	~ keel	-5.923	1.332	4.447	**<0.001**	-0.158
	~ wing	-2.176	0.469	-4.639	**<0.001**	-0.619
Covariances	wing					
	~~ molt	-0.267	0.097	-2.758	**0.006**	-0.441
	~~ wingbeat frequency	-2.461	2.799	-0.879	0.379	-0.344
	mass					
	~~ keel	0.050	0.022	2.301	**0.021**	0.375
Variances	.speed	0.603	0.175	3.452	**<0.001**	0.679
	.load lifting	0.190	0.660	0.289	**0.001**	0.679
	.wing	2.987	0.754	3.962	**<0.001**	0.266
	.BMF	0.064	0.014	4.597	**<0.001**	0.207
	.stroke amplitude	80.336	17.70	4.540	**<0.001**	0.578
	.WBF	17.09	17.426	0.980	0.327	0.469
	molt	0.122	0.027	4.583	**<0.001**	1.000
	tip velocity	0.436	0.522	0.836	0.403	1.00
	keel	0.280	0.061	4.583	**<0.001**	0.215

Script 1

R script to reproduce path models in LAVAAN

```
#### Top speed path analysis

# R version 3.6.3 "Holding the Windsock"

library(lavaan) #v0.6-7
library(semPlot) #v1.1.2
library(readxl) #v1.3.1
library(psych) #v2.0.12

setwd("C:/path/to/spreadsheet/Dataset_1.xlsx")

speed <- read_excel("Dataset_1.xlsx", sheet = "path_data")
speed <- as.data.frame(speed)
speed <- cbind(speed, dummy.code(speed$species))


#### kinematics model
kinematics <- '
wingtip_velocity =~ wing + SA + WBF
speed_3 ~ wingtip_velocity
lifted_3 ~ WBF + SA
wing + mass ~ sex+COHU+ALHU+BCHU
speed_3 ~~ lifted_3 + molt
beads_3 ~~ mass + wing
wing ~~ molt + WBF
SA ~~ mass
'

kinematicsfit <- sem(kinematics, data=speed)
summary(kinematicsfit, standardized=TRUE, rsquare=TRUE, modindices=FALSE,
fit.measures=TRUE)
fitmeasures(kinematicsfit, c("cfi", "rmsea", "srmr"))
AIC(kinematicsfit)
semPaths(kinematicsfit, what="stand", layout="spring", style="ram", whatLabels="stand",
nCharNodes=0, rotation=1, curvePivot=TRUE, theme="colorblind")


#### muscle model
muscle <- '
speed_3 ~ keel
speed_3 ~~ lifted_3 + molt
beads_3 ~ keel + wing
mass ~~ keel + lifted_3
wing ~~ molt
wing + mass + keel ~ sex+COHU+ALHU+BCHU
'
```

Journal of Experimental Biology • Supplementary information

```
musclefit <- sem(muscle, data=speed)
summary(musclefit, standardized=TRUE, rsquare=TRUE, modindices=FALSE, fit.measures=TRUE)
fitmeasures(musclefit, c("cfi", "rmsea", "srmr"))
AIC(musclefit)
semPaths(musclefit, what="stand", layout="spring", style="ram", whatLabels="stand",
nCharNodes=0, rotation=1, curvePivot=TRUE, theme="colorblind")


#### post-hoc combined model
combined <- '
speed_3 + lifted_3 ~ wingtip_velocity + keel
wingtip_velocity =~ wing + SA + WBF
SA ~ keel + wing
wing ~~ molt + WBF
keel ~~ mass
wing + mass + keel ~ sex+COHU+ALHU+BCHU
'
combinedfit <- sem(combined, data=speed)
summary(combinedfit, standardized=TRUE, rsquare=TRUE, modindices=FALSE,
fit.measures=TRUE)
fitmeasures(combinedfit, c("cfi", "rmsea", "srmr"))
AIC(combinedfit)
semPaths(combinedfit, what="stand", layout="spring", style="ram", whatLabels="stand",
nCharNodes=0, rotation=1, curvePivot=TRUE, theme="colorblind")
```

Dataset 1. Spreadsheet with raw data inputs for Experiment 1 (GLM) and Experiment 2 (path modeling).

Available for download at
https://journals.biologists.com/jeb/article-lookup/doi/10.1242/jeb.251012#supplementary-data

Journal of Experimental Biology • Supplementary information

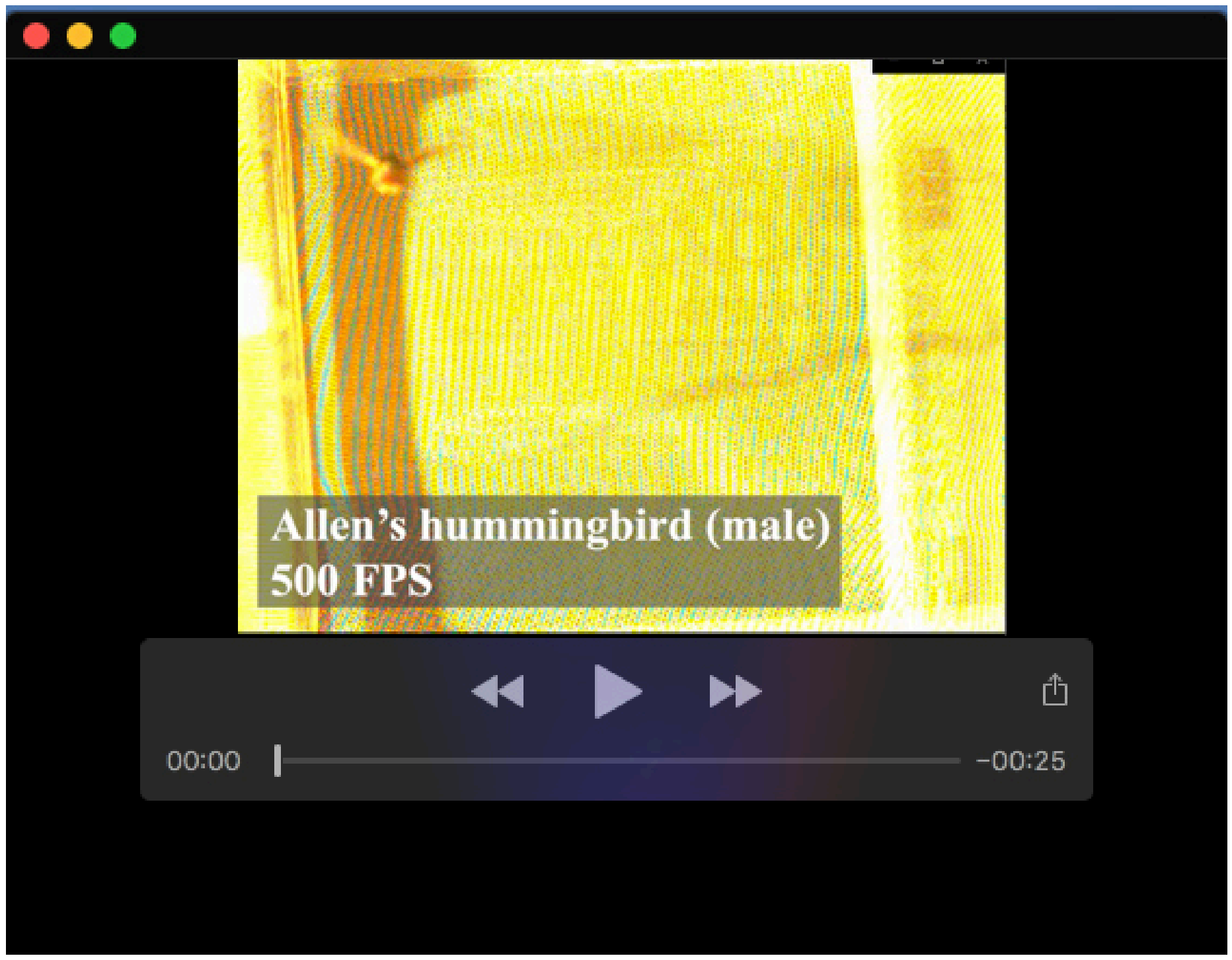

Movie 1. Two examples of wingbeat kinematics of hummingbirds flying at top speed. The first bird is a male Allen's hummingbird. Note the high stroke amplitude. The second bird is a female Costa's hummingbird. Both are filmed at 500 frames per second.

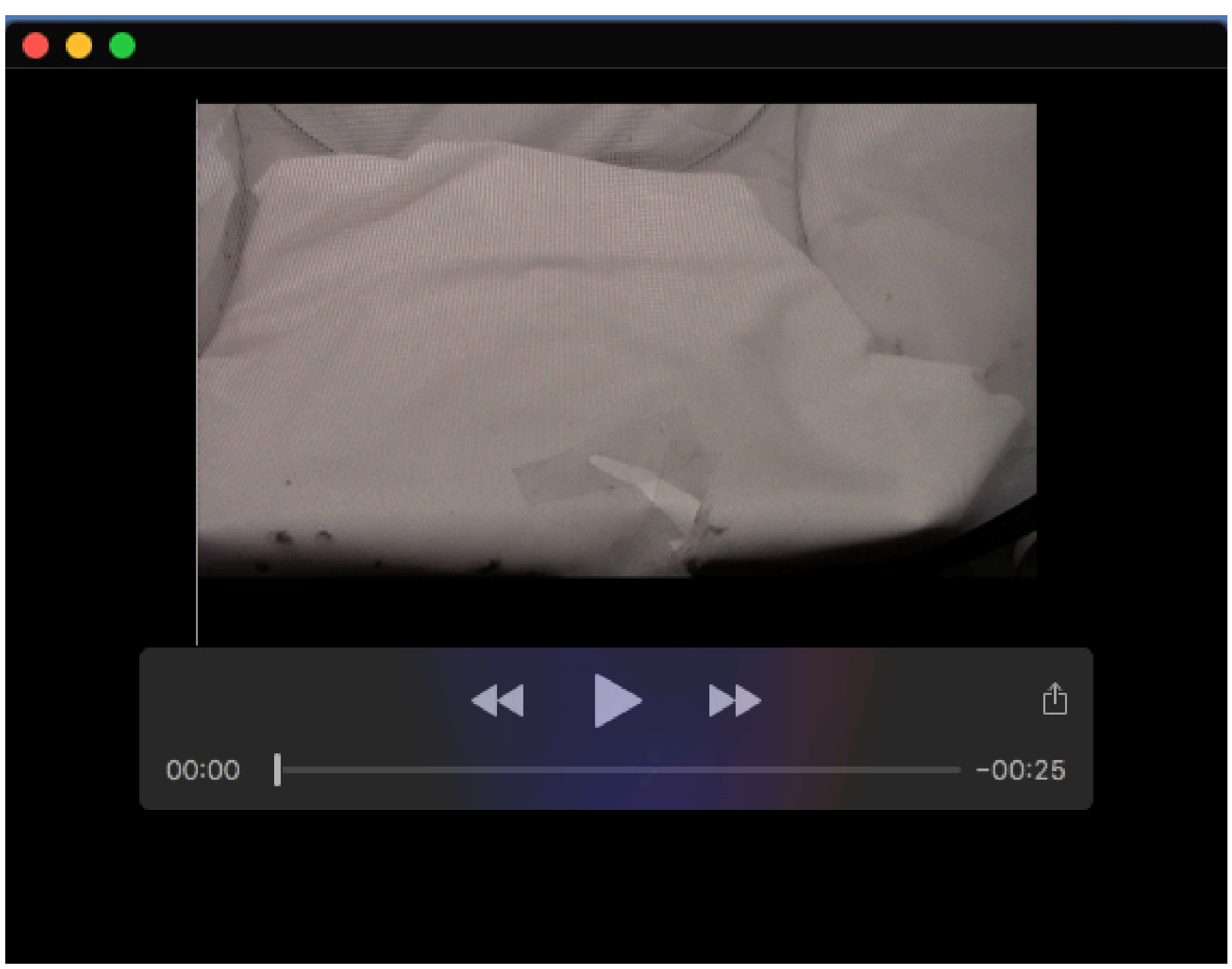

Movie 2. Example load lifting trial framed at 30 frames per second. First the video plays at normal speed, then slows down to indicate which bead was the last lifted.

Journal of Experimental Biology • Supplementary information
